# Pharmacokinetic variability and exposures of fluconazole, anidulafungin, and caspofungin in intensive care unit patients: Data from multinational Defining Antibiotic Levels in Intensive care unit (DALI) patients Study

**DOI:** 10.1186/s13054-015-0758-3

**Published:** 2015-02-04

**Authors:** Mahipal G Sinnollareddy, Jason A Roberts, Jeffrey Lipman, Murat Akova, Matteo Bassetti, Jan J De Waele, Kirsi-Maija Kaukonen, Despoina Koulenti, Claude Martin, Philippe Montravers, Jordi Rello, Andrew Rhodes, Therese Starr, Steven C Wallis, George Dimopoulos, on behalf of the DALI Study authors

**Affiliations:** Burns Trauma and Critical Care Research Centre, The University of Queensland, Brisbane, Australia; School of Pharmacy and Medical Sciences, University of South Australia, Adelaide, Australia; Therapeutics Research Centre, Basil Hetzel Institute for Translational Health Research, The Queen Elizabeth Hospital, Adelaide, Australia; Royal Brisbane and Women’s Hospital, Brisbane, Australia; School of Medicine, Hacettepe University, Ankara, Turkey; Azienda Ospedaliera Universitaria Santa Maria della Misericordia, Udine, Italy; Ghent University Hospital, Ghent, Belgium; Helsinki University Central Hospital, Helsinki, Finland; Attikon University Hospital, Athens, Greece; Hospital Nord, Marseille, France; AzuRea Group, Antibes, France; Centre Hospitalier Universitaire Bichat-Claude Bernard, AP-HP, Université Paris VII, Paris, France; CIBERES, Vall d’Hebron Institute of Research, Universitat Autonoma de Barcelona, Barcelona, Spain; St George’s Healthcare NHS Trust and St George’s University of London, London, England

## Abstract

**Introduction:**

The objective of the study was to describe the pharmacokinetics (PK) of fluconazole, anidulafungin, and caspofungin in critically ill patients and to compare with previously published data. We also sought to determine whether contemporary fluconazole doses achieved PK/pharmacodynamic (PD; PK/PD) targets in this cohort of intensive care unit patients.

**Methods:**

The Defining Antibiotic Levels in Intensive care unit patients (DALI) study was a prospective, multicenter point-prevalence PK study. Sixty-eight intensive care units across Europe participated. Inclusion criteria were met by critically ill patients administered fluconazole (*n* = 15), anidulafungin (*n* = 9), and caspofungin (*n* = 7). Three blood samples (peak, mid-dose, and trough) were collected for PK/PD analysis. PK analysis was performed by using a noncompartmental approach.

**Results:**

The mean age, weight, and Acute Physiology and Chronic Health Evaluation (APACHE) II scores of the included patients were 58 years, 84 kg, and 22, respectively. Fluconazole, caspofungin, and anidulafungin showed large interindividual variability in this study. In patients receiving fluconazole, 33% did not attain the PK/PD target, ratio of free drug area under the concentration-time curve from 0 to 24 hours to minimum inhibitory concentration (*f*AUC_0–24_/MIC) ≥100. The fluconazole dose, described in milligrams per kilogram, was found to be significantly associated with achievement of *f*AUC_0–24_/MIC ≥100 (*P* = 0.0003).

**Conclusions:**

Considerable interindividual variability was observed for fluconazole, anidulafungin, and caspofungin. A large proportion of the patients (33%) receiving fluconazole did not attain the PK/PD target, which might be related to inadequate dosing. For anidulafungin and caspofungin, dose optimization also appears necessary to minimize variability.

**Electronic supplementary material:**

The online version of this article (doi:10.1186/s13054-015-0758-3) contains supplementary material, which is available to authorized users.

## Introduction

The epidemiology of invasive fungal infections (IFIs) in intensive care units (ICUs) is shifting away from those patients traditionally considered at risk because of advances in diagnostic and therapeutic interventions [1]. *Candida* spp. are the third leading cause of infections in ICUs globally, accounting for up to 90% of all fungal infections [2]. *C. albicans* is still the leading cause of fungal infections in ICUs, accounting for 40% to 60% of all invasive *Candida* infections [3]. However, over recent decades, the incidence of *C. albicans* infections has decreased, with a relative increase in non-*albicans Candida* spp. infections, with further geographic variations also present [3,4]. In the critically ill, invasive *Candida* spp. infections are associated with high crude and attributable mortalities as high as 60% and 40%, respectively [1]. In contrast to *Candida* spp. infections, the incidence of *Aspergillus* spp. infections ranges from 0.3% to 6.9% in ICU patients and accounts for up to 7% of all fungal infections. Although the incidence is not as high as that of *Candida* spp. infections, invasive aspergillosis is a debilitating infection with mortality rates as high as 90% being reported [5].

Fluconazole and echinocandins are the most commonly used antifungal agents in ICUs for invasive fungal infections. Therapeutic preference of these antifungal agents often depends on the local epidemiology, type of therapy (prophylaxis, empiric, preemptive, or definitive) and clinical status of the patient. Fluconazole is recommended for prophylaxis against invasive candidiasis in ICU patients with abdominal surgery, recurrent gastrointestinal perforations, or anastomotic leakages. It is also recommended for the treatment of candidemia in less critically ill patients (Acute Physiology and Chronic Health Evaluation (APACHE) II score <15) without recent exposure to azole antifungal agents [6,7]. Fluconazole exhibits concentration- and time-dependent antifungal activity with a prolonged post-antifungal effect. In line with this, the ratio of free drug area under the concentration-time curve from 0 to 24 hours to minimum inhibitory concentration (*f*AUC_0–24_/MIC) is considered the predictive pharmacokinetic (PK)/pharmacodynamic (PD; PK/PD) index associated with maximal fungal killing. An *f*AUC_0–24_/MIC of at least 100 is associated with fungicidal activity and optimal outcomes in the clinical setting, especially when treating immunocompromised or critically ill patients [8].

The addition of echinocandins (for example, anidulafungin and caspofungin) to the antifungal armamentarium was an important step forward in the treatment of invasive candidiasis, considering the increasing prevalence of non-*albicans Candida* spp. infections, especially *C. glabrata*, resistant to fluconazole. Echinocandins are recommended as a first-line treatment option for invasive *Candida* spp. infections in hemodynamically unstable nonneutropenic patients with prior exposure to azoles [6,7]. Antifungal activity of echinocandins has been correlated with both ratio of peak plasma concentration to MIC (C_max_/MIC) and AUC_0–24_/MIC [9]. However, to date, a robust PK/PD index for anidulafungin and caspofungin relating exposure to response is yet to be identified in clinical studies.

Inadequate initial antifungal dosing contributes not only to suboptimal outcomes [10-12] but also to the emergence of resistance [13]. In addition, critically ill patients tend to have an array of pathophysiological changes that can cause antifungal PK alterations, potentially leading to subtherapeutic exposures [14]. Thus, doses established based on the experience from other patient cohorts may not always be optimal to treat patients in ICU, as evidenced for antibacterial agents [15]. Therefore, it is important to understand the disposition of the frequently used antifungal agents like fluconazole, anidulafungin, and caspofungin in critically ill patients.

The objective of the study was to describe the PK of fluconazole, anidulafungin, and caspofungin in critically ill patients and to compare with previously published data. We also sought to determine whether contemporary fluconazole doses achieve PK/PD target in this cohort of ICU patients.

## Methods

### Study design

The Defining Antibiotic Levels in Intensive care unit patients (DALI) study was a prospective, multicenter PK point-prevalence study. The protocol has been published in detail previously [16], and the participating investigators are listed in Additional file 1). The antifungal agents that were included in the DALI Study were fluconazole, anidulafungin, and caspofungin.

### Study population

All ICU patients at participating sites were screened, and eligible patients were identified for participation in the study by the clinical staff on the Monday of the nominated week. Subsequently, blood sampling and data collection occurred throughout the nominated week. The lead site was The University of Queensland, Australia, with ethical approval granted by the Medical Research Ethics Committee (Number 201100283, May 2011). All participating centers in the DALI Study obtained ethics approval from their respective ethics committees (Additional file 2). Informed consent was obtained for each eligible patient.

### Drug administration and sample collection

The choice of antifungal agent and dosing was at the discretion of the treating clinician. Each patient had three blood samples taken: blood sample A was a peak concentration taken 30 minutes after completion of intravenous infusion; blood sample B was a mid-dose blood sample taken 50% of the way through a dosing interval; and blood sample C was a pre-dose concentration taken at the end of a dosing interval (within 30 minutes of the next dose).

Blood samples were processed and stored per protocol to maintain sample integrity. A commercial courier company transported the clinical samples on dry ice to the coordinating center (Burns, Trauma and Critical Care Research Centre, The University of Queensland, Australia).

### Data collection

Data collection was performed by trained staff at each participating center and entered into a case report form. Various demographic and clinical data were collected, including age, gender, height, weight, admission diagnosis, presence of extracorporeal circuits (for example, renal replacement therapy), clinical outcome of infection, and mortality at 30 days. A positive clinical outcome of therapy was defined as completion of treatment course without change or addition of antifungal therapy and with no additional antifungals commenced within 48 hours of discontinuation of the therapy, and if the outcome of therapy could not be judged for any reason, it was recorded as indeterminate. Also, organ-function data (including renal function: serum creatinine concentration during studied dosing interval; 24-hour measured urinary creatinine clearance (CrCL) (where available), antibiotic dosing (dose and frequency, time of dosing and sampling, day of antibiotic therapy), and infection data (including known or presumed pathogen and pathogen MIC) were collected.

Antibiotic dosing data including the dose (in milligrams and in milligrams per kilogram total body weight), infusion duration, frequency of administration, the time of dosing and sampling, and the day of antibiotic therapy were collected. All data were collated by the coordinating center.

### Sample analysis

Anidulafungin (0.5 to 0 μg/ml), caspofungin (0.1 to 20 μg/ml), and fluconazole (0.2 to 20 μg/ml) were measured in plasma by separate validated UHPLC-MS/MS methods on a Shimadzu Nexera system coupled to a Shimadzu 8030+ triple quadrupole mass spectrometer (Shimadzu Corporation, Nakagyo-ku, Kyoto, Japan). The stationary phase was a Kinetex C8 (50 × 2.10 mm, 1.7 μm) UHPLC column (Phenomenex, Torrance, CA, USA). The mobile phase was a gradient of acetonitrile and 0.1% formic acid (anidulafungin and caspofungin) or acetonitrile and 0.1% formic acid with 10 m*M* ammonium formate (fluconazole). Ionization was by positive-mode electrospray, with analytes detected at the following MRMs: 1140.4 → 343.10 (anidulafungin), 1,093.50 → 1,033.50 (caspofungin), 306.7 → 238.2 (fluconazole), 470.10 → 160.20 (dicloxacillin), and 350.0 → 281.1 (voriconazole). Plasma (100 μl) was spiked with internal standard (dicloxacillin for anidulafungin/caspofungin, and voriconazole for fluconazole) and treated with acetonitrile to precipitate proteins before instrumental analysis.

For anidulafungin analysis, the supernatant was washed with dichloromethane to remove lipid-soluble components. Sample analysis met batch acceptance criteria. The methods were validated for linearity, LLOQ, matrix effects, precision and accuracy, and stock stability by using the FDA criteria for bioanalysis [17].

### PK analysis

The PK parameters for each drug were estimated by using noncompartmental methods. The apparent terminal elimination-rate constant (k_el_) was determined from log-linear least-squares regression. The minimum and the peak concentration for the dosing period (C_min_ and C_max_) were the observed values. Half-life (T_1/2_) was calculated as ln (2)/k_el_. The area under the concentration-time curve from 0 to 24 hours (AUC_0–24_) was calculated by using the linear trapezoidal approximation. The area under the plasma concentration–time curve from time 0 to infinity (AUC_0–inf_) was calculated by the log-linear trapezoidal rule until the time of last quantifiable plasma concentration and then extrapolated to infinity by using the quotient of the last measurable concentration to the terminal phase rate constant (k_el_). The AUC from 0 to 24 hours (AUC_0–24_) was calculated by using a doubling of AUC_0–12_, assuming steady state when fluconazole was administered every 12 hours. Clearance (CL) was calculated by using the equation Dose/AUC_0-∞_. Protein binding of fluconazole was assumed to be 12 to determine free AUC_0–24_ (*f*AUC_0–24_) [18]. For fluconazole, *f*AUC_0–24_/MIC of at least 100 was chosen as the target PK/PD index associated with efficacy [8].

## Results

### Fluconazole

Fluconazole blood samples were collected from 26 ICU patients. Eleven patients were excluded, as the PK parameters or *f*AUC_0–24_/MIC could not be estimated from the available data. As such, the study sample consisted of 15 patients from 12 ICUs in six countries (Belgium, Finland, France, Greece, Portugal, and Turkey). In 67% of the patients, fluconazole samples were collected at least 5 days post-initiation of treatment. Measured CrCL was available for only 10 patients. Demographic and clinical data, including disease severity and CrCL, are described in Table 1. *C. albicans* was isolated from six patients, of whom MIC was reported in one case (MIC = 0.75 mg/L), *C. glabrata* was isolated from one patient, and in the remaining patients, fungal pathogen was not isolated. Of patients administered fluconazole, 27% (*n* = 4) achieved a positive clinical outcome, with clinical outcome described as indeterminate in 40% (*n* = 6) of patients; 47% (*n* = 7) of patients had died by day 30.

**Table 1:** **Demographics, clinical characteristics, and dose in critically ill patients receiving fluconazole, anidulafungin, and caspofungin therapy**

	**Fluconazole**	**Anidulafungin**	**Caspofungin**
**Number of patients**	15	9	6
**Age (years)**	56 (44–82)	51 (41–66)	62 (58–72)
**Weight (kg)**	82 (80–90)	81 (76–92)	80 (75–85)
**Male sex**	11 (73)	7 (78)	5 (71)
**Dose received (mg)**	400 (200–400)	100	70 (loading dose)
**Dose (mg/kg)**	4.9 (2.3–5.0)	NA	NA
**APACHE II score**	22 (10–30)	18 (15–32)	22 (20–26)
**SOFA score**	7 (5–9)	6 (3–7)	3 (2–8)
**Serum creatinine concentration (m** ***Μ*** **)**	88 (64–203)	119 (47–228)	258 (117–286)
**Measured creatinine clearance (ml/min)**	45 (14–103)	62 (15–113)	NA

Data are described as median [1st to 3rd quartile] or *n* (%).

APACHE, Acute Physiology and Chronic Health Evaluation II score; SOFA, sequential Organ Failure Assessment; NA, insufficient patients with measured creatinine clearance/not applicable.

**Figure 1:**
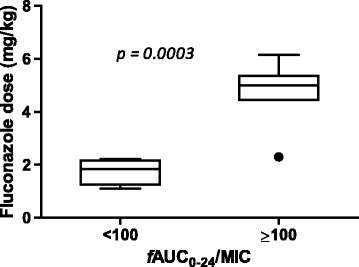
**The box plot of fluconazole dose in milligrams per kilogram stratified by the patients achieving and not achieving the PK/PD index, free drug area under the concentration-time curve from 0 to 24 hours (** ***f*** **AUC**_**0–24**_ **/MIC).**

As shown in Table 1, fluconazole median dose was 400 mg (4.9 mg/kg). The observed fluconazole AUC_0–24_, C_max_, and C_min_ in comparison with other studies is presented in Table 2. The target *f*AUC_0–24_/MIC ≥100 was reached in 86% of patients when the assumed MIC was 1 mg/L. If MIC 2 mg/L (clinical breakpoint) and 4 mg/L (clinical breakpoint for resistance by using EUCAST methods and susceptible dose-dependent by using CLSI methods) were used, then the target *f*AUC_0–24_/MIC ≥100 was reached in 67% and 13% of patients, respectively. As expected, dose was found to be significantly associated with the achievement of *f*AUC_0–24_/MIC ≥100 (*n* = 15, *P* = 0.0003) (Figure 1). The median dose received in patients with *f*AUC_0–24_/MIC ≥100 was 5 mg/kg compared with 2 mg/kg in patients with *f*AUC_0–24_/MIC <100. Only two (20%) patients, both with CrCL < 50 ml/min, received the recommended weight-based fluconazole dose and both attained the target exposure. No difference was observed in daily doses between patients with CrCL <50 ml/min (median (1st to 3rd quartile) to 2.3 (1.7 to 5.0) mg/kg) and with CrCL >50 ml/min (4.4 (1.8 to 5.0) mg/kg) (*P* = 0.65).

**Table 2:** **Comparison of exposures achieved in this study with other studies (mean (%CV))**

	**Anidulafungin**			**Caspofungin**			**Fluconazole**		
**Parameter**	**This study (ICU patients)**	**Liu et al.** [25] **(ICU patients)**	**Liu et al.** [25] **(healthy volunteers)**	**This study** **(ICU patients)**	**Wurthwein et al.** [26] **(general patients)**	**Stone et al.** [27]^**a**^ **(healthy volunteers)**	**This study** ^**b**^ **(ICU patients)**	**Buijk et al.** [23]^**a**^ **(surgical patients)**	**Sobue et al.** [24]^**b**^ **(Healthy volunteers, 800 mg)**
**AUC**_**0–24**_	55 (28)	93.0 (41)	105.0 (22)	52.0 (53)	170.0 (34)	97.0 (87–109)	359 (259)	409 (336–482)	608 (118)
**C**_**max**_	3.9 (29)	7.7 (56)	7.0 (22)	3.9 (55)	13.8 (31)	12.1 (11–13)	20 (14)	25 (22–28)	34 (6)
**C**_**min**_	1.8 (30)	3.0 (44)	3.1 (25)	1.5 (57)	4.2 (2.56)	1.4 (1.1–1.8)	14 (11)	15 (10–20)	20 (NR)

^a^Mean (95% CI); ^b^Mean (SD). AUC_0–24_, area under the concentration-time curve from 0 to 24 hours C_max_, observed maximum concentration; C_min_, observed minimum concentration; CV, coefficient of variation; ICU, intensive care unit.

### Anidulafungin and caspofungin

Anidulafungin blood samples were collected from 10 ICU patients, with one patient excluded as PK parameters could not be estimated from available data. As such, the study sample consisted of nine patients from seven ICUs of three countries (Spain, Italy, and Greece). Caspofungin blood samples were collected from seven patients from six ICUs of five countries (Belgium, France, Greece, Italy, and Portugal). Demographic data, disease severity, and renal function are described in Table 1. *C. albicans* was the isolated pathogen in the majority of the cases (six patients), with *Aspergillus* spp. isolated from two patients receiving caspofungin. In terms of clinical outcome for the anidulafungin patients, 67% (*n* = 6) were defined to have achieved a positive clinical outcome, and 33% (*n* = 3) had an indeterminate clinical outcome. For the caspofungin patients, 43% (*n* = 3), achieved a positive clinical outcome, and 11% (*n* = 3) had an indeterminate clinical outcome. The 30-day mortality was 11 (*n* = 1) and 29% (*n* = 2) for the patients administered anidulafungin and caspofungin, respectively. The observed anidulafungin and caspofungin AUC_0–24_, C_max_, and C_min_ in comparison to other studies are presented in Table 2.

## Discussion

This is the first multinational multicenter point-prevalence study reporting PK variability of fluconazole, anidulafungin, and caspofungin, and PK/PD target attainment of fluconazole in critically ill patients. This study demonstrated that 33% of patients receiving fluconazole did not achieve the desired PK/PD index required for optimal outcome. In light of the increasing information on the antifungal exposure/response relation [12,19-22], achieving the PK/PD target should be considered critical to improve the patient outcomes and to minimize the emergence of fluconazole resistance.

The observed fluconazole AUC_0–24_, C_min_, and C_max_ were comparable to values previously reported in surgical ICU patients [23]. However, variability was higher compared with healthy volunteers (Table 2) [24]. This may be attributed to underlying pathophysiological changes that occur in ICU patients. The observed anidulafungin variability in AUC_0–24_, C_min_, and C_max_ was not as high as observed in the study by Liu *et al.* [25], who recruited a larger cohort of surgical ICU patients but was higher than variability observed in healthy volunteers (Table 2) [25]. The PK parameter estimates were also lower than those observed by Liu et al. [25]. The patient cohort in our study were younger (60 vs. 51 yrs) with a higher median weight (65 vs. 81 kg) compared to the cohort in the study by Liu et al. [25]. It is possible that the increased clearance associated with these differences may explain the lower exposures observed in this study, although these postulates are yet to be proven for anidulafungin. For caspofungin, the observed variability was higher than that reported in patients with invasive aspergillosis with lower exposures compared with invasive aspergillosis patients and healthy volunteers [26,27] (Table 2). However, the C_min_ observed in the current study was comparable to that observed by Nguyen et al. [28] in surgical ICU patients with large variability (0.5 to 4.1 mg/L versus 0.5 to 2.6 mg/L, in this study). The reasons for lower exposures observed for caspofungin in our study may be two-fold – firstly, 70% of patients were sampled on day 1 and secondly, our patient cohort was critically ill compared to surgical patients and patients with invasive aspergillosis in other studies [27,29]. Shock in particular may be a strong factor given that in an experimental shock model in pigs, a 25% reduction in AUC was observed during the initial shock phase [30].

An important observation from this study was the variation in doses of fluconazole used (Table 1). In particular, use of a “standard” 400-mg daily dose was common, which when converted to a mg/kg dose, commonly fell below the 6 mg/kg recommended dose [7] (with 12 mg/kg loading dose), which was then associated with not achieving the PK/PD target (Figure 1). Although a 50% dose reduction is recommended in patients with CrCL <50 ml/min [29], neither these patients with renal dysfunction nor those with normal renal function had a median dose in line with the recommended dose. Of note, only two (20%) patients, both with CrCL <50 ml/min, received the recommended weight-based fluconazole dose, and both attained the target exposure. Our data support observations from previous studies, which have demonstrated that suboptimal dosing with fluconazole is prevalent [30]. Moreover, as shown in Figure 1, a clear association exists between the mg/kg dose administered and attainment of a PK/PD target supporting individualized weight-based dosing. Indeed, the use of the standard 400-mg dose appears to result in suboptimal exposure, suggesting that a “one dose fits all” approach for fluconazole is flawed. It follows that in some patients with higher body weight, doses higher than the standard 400 mg are required. In line with this, the use of loading doses may help the early achievement of PK/PD targets for fluconazole. Toxicity concerns should not dissuade clinicians from more-aggressive doses because fluconazole is a very-well-tolerated drug with much higher doses successfully used without adverse effects [31].

We could not attempt to determine the PK/PD target attainment for anidulafungin and caspofungin, as the PK/PD targets relating exposure to clinical response are yet to be defined, and data from animal studies were found to be highly variable.

Some limitations of this study were found. First, although many ICUs were included in the study, the number of recruited patients was not comprehensive, and therefore, we may be underestimating the actual PK variability in the population. Despite this, the recruitment of patients from many different ICUs in different countries does enhance the generalizability of the results.

Second, we did not measure unbound concentrations of the antifungals, although it should be noted that previous studies defining PK/PD targets have also measured total drug concentrations.

Third, this study used only three concentrations to estimate PK parameters, although this is likely to be appropriate, given the relatively long half-life of these drugs. Finally, we used breakpoint MICs determined by EUCAST and CLSI to estimate fluconazole PK/PD targets because of the lack of reported actual MICs for a large proportion of patients.

## Conclusion

This study demonstrated that contemporary dosing of fluconazole causes a significant proportion of patients not achieving PK/PD targets because of PK variability. For fluconazole, this appears to be caused by the use of a fixed-dosing approach (that is, 400 mg daily) rather than the recommended weight-based dosing regimen. In this study, patients receiving adequate weight-based doses of fluconazole were more likely to achieve PK/PD targets. Similarly, a large variability was observed in PK for anidulafungin and caspofungin. With the limitation of small sample size, this study illustrates that antifungal dosing in the critically ill is as complex as previously demonstrated for antibiotics. Further research is required to optimize exposures of antifungal agents and thus potentially to improve clinical outcomes associated with antifungal use in critically ill patients.

## Key messages

- We have described pharmacokinetic variability of fluconazole, anidulafungin, and caspofungin and PK/PD target attainment of fluconazole in critically ill patients.
- Considerable interindividual variability was observed for fluconazole, anidulafungin, and caspofungin. In anidulafungin and caspofungin, observed exposures were lower than those observed in other ICU studies and patient cohorts.
- Suboptimal dosing with fluconazole is prevalen; 33% of patients receiving fluconazole did not achieve the desired PK/PD index required for optimal outcome. Patients receiving adequate weight-based doses (mg/kg) of fluconazole were more likely to achieve PK/PD targets
- Toxicity concerns should not dissuade clinicians from more-aggressive doses because fluconazole is a very-well-tolerated drug with much higher doses successfully used without adverse effects.
- With the limitation of small sample size, this study illustrates that antifungal dosing in the critically ill is as complex as previously demonstrated for antibiotics. Further research is required to optimize exposures of antifungal agents in at-risk critically ill patients.

## Additional files

Additional file 1:
**A list of the DALI Study Authors.**
Additional file 2:
**A list of the contributing sites and their ethical approval bodies.** Please see the attached Word document with the track changes.

## Abbreviations

APACHE: Acute Physiology and Chronic Health Evaluation
AUC_0–24_: area under the concentration-time curve from 0 to 24 hours
AUC_0–inf_: area under the plasma concentration–time curve from time 0 to infinity
CL: clearance
CLSI: Clinical and Laboratory Standards Institute
C_max_/MIC: ratio of peak plasma concentration to MIC
C_min_: minimum concentration for the dosing period
CrCL: creatinine clearance
EUCAST: European Committee for Antimicrobial Susceptibility and Testing
DALI: Defining Antibiotic Levels in Intensive care unit patients
FDA: Food and Drug Administration
*f*AUC_0–24_/MIC: ratio of free drug area under the concentration-time curve from 0 to 24 hours to minimum inhibitory concentration
T_1/2_: half-life
ICU: intensive care unit
IFI: invasive fungal infection
k_el_: apparent terminal elimination-rate constant
LLOQ: lower limit of quantification
MIC: minimum inhibitory concentration
MRM: multiple reaction monitoring
PD: pharmacodynamic
PK: pharmacokinetic
UHPLC-MS/MS: ultra high performance liquid chromatography tandem mass spectrometry
